# Bad dream, nightmares and psychopathology: a systematic review

**DOI:** 10.3389/fpsyt.2024.1461495

**Published:** 2024-10-08

**Authors:** Julie Faccini, Jonathan Del-Monte

**Affiliations:** ^1^ Laboratory of Clinical, Cognitive and Social Anthropology and Psychology (LAPCOS), University of Cote d’Azur, Nice, France; ^2^ Emotions, Neurocognition and Therapeutic Behavioral Approaches (ENACT) Team, University of Nîmes, Social Psychology Laboratory, Aix-Marseille University, Nîmes, France

**Keywords:** nightmare, bad dream, nightmare disorder, psychiatric disorder, psychopathology

## Abstract

**Objective:**

Bad dreams and nightmares can have a strong psychological impact. However, although the scientific literature points in the direction of an established association between nightmares and psychopathology, many of the studies investigating these links have been carried out on a general population. This systematic literature review aimed to identify studies that have used a sample from a population with a diagnosis of psychopathological disorders or a diagnosis of nightmare disorder, to highlight the state of knowledge concerning the link between bad dreams, nightmares, and psychopathology.

**Method:**

The systematic review included all cross-sectional and longitudinal studies conducted on a psychiatric population in which variables related to bad dreams and/or nightmares were examined and published since 2014. The search was conducted in May 2024 in the PsychINFO and PubMed databases.

**Results:**

A total of 34 studies published over the last decade met the inclusion criteria.

**Conclusions:**

Psychiatric populations are significantly more affected by nightmares and bad dreams than the general population. Furthermore, the presence of nightmares tends to maximize the clinical symptomatology of patients. However, there are still too few studies available to understand the basis of this relationship.

## Introduction

Nightmare disorder is defined in the *Diagnostic and Statistical Manual of Mental Disorders, Fifth Edition, Text Revision* (*DSM-5-TR*) as repeated awakenings with a recollection of terrifying dreams, usually involving threats to survival, safety, or physical integrity (1). Nightmare disorder is to be distinguished from nightmares, which are temporary. Nightmares are a frequent phenomenon in the general population, about 70% (2) where their occurrence is occasional (3). 2-5% of the adult population suffers from frequent nightmares (2). An equal percentage of people report having a “nightmare problem” (2) underpinned by the repetition of nightmares associated with distress. Moreover, nightmares are very often associated with other sleep disturbances such as insomnia (4, 5). Nightmares usually occur during REM sleep (6). They are associated with symptoms of physical arousal such as sweating and shortness of breath, as well as higher indices of periodic leg movements during sleep and rapid eye movements (7). Fear is the emotion most experienced during a nightmare, leading to a sudden awakening. This awakening is one of the conditions that distinguish nightmares from “bad dreams”, although in both cases, fear is also extremely present. Scientific consensus distinguishes nightmares from bad dreams based on the wakefulness engendered by the dream production. However, Spoormaker et al. (8) point out that the need for an alarm clock may be unnecessarily narrow since waking directly from a bad dream is not associated with increased distress. Only post-traumatic nightmares seem to be linked to more frequent and longer night-time awakenings (8). In addition, 45% of bad dreams have an emotional intensity equal to or greater than that of nightmares (9). Bad dreams and nightmares are a source of many strong emotions in addition to fear, such as anger or sadness. The literature tends to divide the content of nightmares into two broad categories: “physical threats” (physical aggression, fighting, murder) and “psychological threats” (abandonment, betrayal, rejection, humiliation) (10–12). These dream contents are also a marker of the difference between idiopathic nightmares and post-traumatic nightmares (PTSD). In PTSD, the contents are a quasi-exact simulation of the traumatic event or emotional sequence experienced (13, 14). PTSD is one of the few psychopathological disorders in which nightmares play a central role and are included in the diagnostic criteria. However, we now know from research that nightmares are closely linked to multiple pathological families. Starting with the etiology of nightmares, which share the same fertile ground for the development of psychopathological disorders. According to Gieselmann’s Integrative model of nightmare etiology (14), several dimensions are at the root of nightmare disorder. The author reports the presence of physiological factors, such as sleep apnea, which promote hyperarousal and hinder the extinction of fear. This condition is strongly associated with mental disorders (15). The author also reports the presence of traumatic experiences, and childhood adversity favoring the emergence of trait-affect distress. In addition, cognitive factors constitute a significant vector in this etiological model, in particular dysfunctional beliefs, thought suppression, and dream rebound. This type of cognition has also been associated as a vulnerability factor in the development of psychopathological disorders such as depression (16) or anxiety disorders (17). Nightmares and psychopathology share some of the same physiological and psychological aspects. At the interface of these two pathways, we find another common pattern: interoception. Interoception refers to the process by which the nervous system senses, interprets, and integrates signals originating from within the body (18). Dysfunction of interoception is increasingly recognized as an important component of different mental health conditions (18). This same component, responsible for homeostatic disruption, has been reported to be associated with nightmares (19, 20). Beyond common patterns, the relationship between nightmares and mental disorders is close, even causal. Indeed, in a recent literature review, Sheaves et al. (21) reported on the effects of specific treatment of nightmares on the expression of psychopathological disorders, and vice versa. The findings point to a causal effect, with studies, albeit few in number, showing a reduction in clinical symptoms when nightmares are specifically treated, and a reduction in the presence of nightmares when the mental disorder is managed.

Although the literature provides solid evidence of an interrelationship between the deleterious expressivity of nightmares and mental disorders, this parasomnia is nonetheless under-considered in clinical practice (22), and also in theoretical understanding of the development and maintenance of mental illness. Indeed, while nightmares are firmly anchored alongside PTSD in our knowledge of the links between sleep disturbances and the psychopathological sphere, the fact remains that the literature has shown that this parasomnia can occupy an important place alongside other disorders.

This literature review aims to shed light on the relationships between bad dreams, nightmares, nightmare disorder, and psychopathological disorders or symptoms, by listing the studies that have investigated the nature of these links in adults. The present study aimed to conduct a systematic review of the literature to describe the relationships and findings of research that has evaluated the links between bad dreams and nightmares with post-traumatic stress disorder, depression, anxiety, schizophrenia, bipolar disorder, suicide, attention deficit hyperactivity disorder, pathological bereavement, and somatoform disorders. We wanted to propose a more exhaustive review of the psychopathological disorders associated with bad dreams and nightmares, but also a review focusing specifically on the pathological expressions of these interrelationships, selecting only studies in clinical populations. Furthermore, we wished to investigate only those studies reporting these relationships outside the question of nightmare treatment to emphasize the morbid reality of these links. Our literature review suggests a broader psychopathological spectrum and exclusivity in clinical population studies. We feel that this choice is essential if we are to report on the psychopathology of nightmares. General population studies can only offer a partial view of the reality of the association between nightmares and psychopathology, and we don’t know to what extent nightmares and bad dreams may be associated with mental health diagnoses. What’s more, the reviews that have included studies reporting on the management of nightmares in psychic disorders cannot determine the extent to which the specificity of nightmare treatment has impacted the reduction of overall symptomatology compared with other ongoing treatments. Furthermore, in this literature review, we have also included bad dreams, reported to be a source of emotional dysregulation (10).

The purpose of this literature review is to shed new and exhaustive light on the associations between bad dreams, nightmares, and psychopathology in clinical populations.

## Methods

### Literature search

An electronic systematic search of PubMed, PsycINFO, and Scopus was performed to identify articles that examined the associations between bad dreams, nightmares, and psychopathology disorders. The keywords used were: Nightmares OR Nightmare disorder OR bad dream OR Parasomnia OR Disturbing dream OR Scary dream associated with Mental health problem OR Mental health disorders OR Psychiatric disorders OR Psychiatric illness OR Posttraumatic stress disorder OR Depression OR Anxiety disorder OR Schizophrenia OR Bipolar disorder OR Suicide OR Borderline personality disorder OR Attention-deficit hyperactivity disorder OR Pathological grief OR Pathological bereavement. A total of 78 keyword combinations were used. The article search was conducted between 21 May 2024 and 11 August 2024.

### Selection and data extraction

Titles and abstracts were reviewed according to the inclusion and exclusion criteria for the first selection. A second review of the full-text articles was carried out to extract data from the studies and re-check compliance with the inclusion criteria. The articles were selected according to Foster’s & Jewell (23) recommendations.

Study quality was assessed using a series of evaluation questions designed to meet the needs of this review. Each article was assessed according to the quality of the response provided based on the study design with the primary objective of assessing the association between bad dreams, nightmares, and/or nightmare disorder and psychopathological disorders or symptoms outside the consideration of nightmare treatment. We paid particular attention to validated measures of psychiatric symptoms and diagnoses, as well as the measurement of nightmares. We also paid close attention to journal quality, selecting only studies published in peer-reviewed journals. We considered studies providing quantitative and qualitative analyses (used notably in analyzing nightmare content). We have selected studies on the mental pathologies most commonly associated with the literature (posttraumatic stress disorder, depression, anxiety disorder, schizophrenia, bipolar disorder, suicide) as well as those that are coming increasingly to the fore in terms of their links with nightmares and bad dreams (borderline personality disorder, attention-deficit hyperactivity disorder, pathological bereavement).

Data extracted from the studies included: author, year of publication, country, population, sample size, measures of nightmares, measures of psychopathological or other disorders/symptoms (task or sleep disorders), and main results.

### Inclusion criteria

The studies were specifically concerned with the relationship before treatment between bad dreams, nightmares, nightmare disorders, and psychopathological disorders or symptoms.

Any type of study design that investigated the association between bad dreams, nightmares, and psychopathological disorders was considered. Potential study designs included cross-sectional, longitudinal, prospective, retrospective, interventional, and experimental. The selected studies had to have been written in English, published in a peer-reviewed journal, and had to have been published from 2014 to the present day.

Studies using both quantitative and qualitative data were included.

The studies selected had to involve an adult population aged 18 or over. Studies including participants over 60 years of age were considered, provided this age group was not exclusive to the sample. Only studies involving a clinical population, i.e. with a proven diagnosis or meet diagnostic criteria according to standardized measurements: posttraumatic stress disorder, depression, anxiety disorder, schizophrenia, bipolar disorder, suicide risk, borderline personality disorder, attention-deficit hyperactivity disorder, pathological grief, pathological bereavement or nightmare disorder or studies on the interrelationship between nightmare disorder and psychopathological symptoms.

Regarding assessing bad dreams and/or nightmares, studies had to have used either standardized scales, diaries, or interviews with no specific duration required. The elements of the dreams studied could concern frequency, content, and repercussions (stress, distress, etc.).

The selected studies were based exclusively on a clinical population, and the diagnosis had to have been made by a psychiatrist using standardized tests and/or clinical interviews.

### Exclusion criteria

Studies were excluded if the population concerned participants under 18 years of age or only participants over 60 years of age and participants did not meet the criteria for a psychopathological disorder or nightmare disorder. We did not include children and adolescents, because their psychopathological patterns differ from those of adults. Similarly, people over 60 are more likely to have neurocognitive frailties that may be a biological cause of nightmares and psychopathological symptoms.

Articles concerning studies on the evolution of the interrelations between bad dreams, nightmares, and psychopathological disorders during drug or non-drug treatment were not included. Similarly, studies on the evolution of the interrelations between bad dreams, nightmares, and psychopathological disorders during drug or non-drug treatment were not included.

Articles not available in full-text, reviews, meta-analyses, case studies, and studies not written in English and/or published in a peer-reviewed journal were omitted.

### Methodological quality assessment

The two reviewers (JF and JDM) independently appraised the quality of the final set of studies, using the STROBE checklist for cross-sectional and mixed studies (24) and on the CASP checklist for qualitative studies (25). A score was assigned to each article selected and reported in **Supplementary Table 1** after consensus between the authors. The STROBE checklist comprises 22 items assessing the presence or absence of information in the study and generates a score of 0 or 1 per item, i.e. a maximum possible score of 22. The CASP checklist includes 10 items where evaluators can report “Yes” (= 1 point), “Can’t tell” (=0), or “No” (0). The maximum score is 10.

## Results

**Figure 1:**
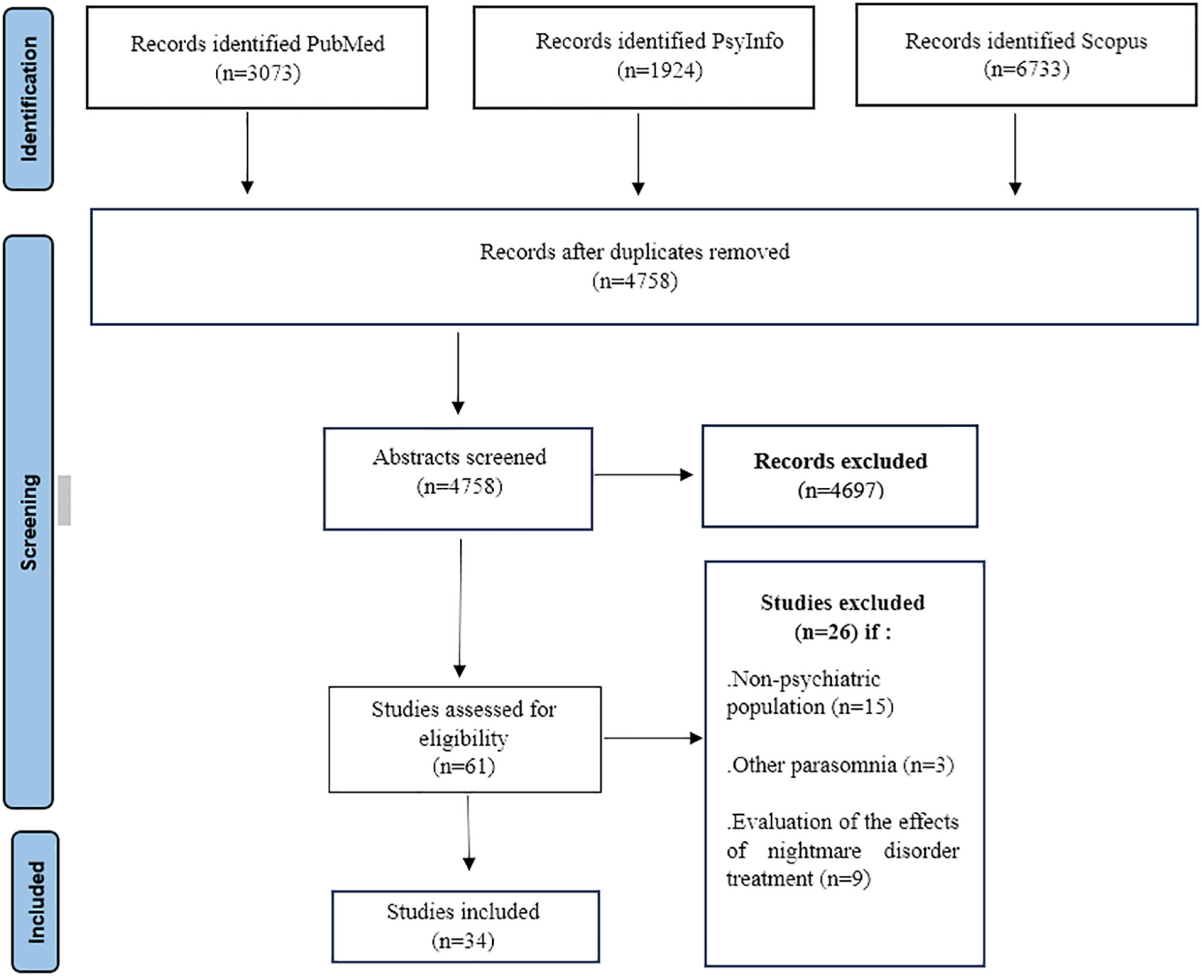
Study flow diagram of selected studies (PRISMA).

The search generated 11730 results. 61 abstracts were retained after removing duplicates (n=605) and after reviewing the abstracts. After a review of the full text, 34 were finally retained (**Figure 1**). The most frequent reasons for elimination were the studies were carried out on a general population, the “parasomnia” measure not allowing the nightmare measure to be distinguished from other parasomnia measures in the results or studies aimed at investigating the management of nightmares or the evolution of nightmares in the treatment setting.

The majority of studies used validated questionnaires and scales to measure clinical, sleep, and other variables (n=27). To assess dreams, studies have used psychometric scales (n=18), specific items (n=7), questionnaires (n=2), visual analog scales (n=1), and interviews (n=5) or dream/sleep report diaries (=9). The quality of the selected studies is very good overall, and the shortcomings identified in a few studies mainly concern a lack of completeness in terms of recruitment and participant characteristics.

Of the 34 studies selected, 34 involved a psychiatric population and 2 involved a population with specific nightmare disorder. Among the studies involving a psychiatric population, 11 studies reported multiple psychiatric symptoms or diagnosis outcomes: 14 with a diagnosis of post-traumatic stress disorder, 10 with depressive disorder, 8 with anxiety disorders (social phobia, specific phobia, general anxiety disorder), 4 with bipolar disorder, 6 with either a diagnosis of schizophrenia, schizophrenic spectrum disorder or psychotic symptoms, 3 with a population at risk of suicide, 3 with a personality disorder, 1 involved a population with a diagnosis of attention deficit disorder with or without hyperactivity and 2 involved a population with nightmare disorder. (**Supplementary Table 1**).

The majority of studies were cross-sectional (n=32). 1 study was longitudinal (8 years) and 2 were short-term (21 days; 3 days).

### PTSD

Studies examining the relationship between bad dreams, nightmares, and post-traumatic stress disorder were the most numerous (n=14). 10 studies focused exclusively on a population with no disorders other than PTSD.

Several studies (n=4) focused on combatants or veterans of the armed forces. Miller et al. (26) conducted a study using in-longitudinal and ambulatory methods to predict morning reports of nightmares and disruptive dreams in 31 (only men) war veterans with PTSD. 468-morning reports (indicating the presence or absence of nightmares) were combined these reports with predictive factors, including the previous night’s mattress actigraphy, indicating heart rate, and respiratory sinus arrhythmia. The authors conducted binary logistic regression models to examine predictors of a morning nightmare report. Results showed that a high Respiratory Event Index (REI) was significantly associated (OR = 1.65, p = .02) with nightmare reports, as was lower sleep-period respiratory sinus arrhythmia (RSA) (OR = 0.69, P = .01). The authors also reported a marginal effect for negative mood on the previous day (OR = 1.32, P = 0.08), with higher scores predicting a greater likelihood of morning nightmares. This study combines both objective and physiological data to account for the interrelationship of nightmares in PTSD and mood. Mader et al. (27) found significant positive associations between the frequency of post-traumatic nightmares and the measures of heart rate, frequency of post-traumatic nightmares, and skin conductance. They also showed that the measures of heart rate significantly predict post-traumatic nightmares. This work is important to complement the growing body of research on nightmares, psychopathology, and interoception. Miles et al. (28) also used objective measures with military personnel suffering from PTSD. The study aimed to better understand how sleep problems and sleep architecture are related to PTSD symptoms. To do this, the authors used a 7-day dream diary, an insomnia scale, and polysomnography. Multiple linear regressions showed that nightmare severity (β: 0.24: p. = 0.04) and frequency (β: 0.26: p. = 0.01) predicted increased PTSD symptom severity. Nightmare severity significantly predicted anger severity which is a residual symptom of PTSD (β: 0.30: p. = 0.01).) Of the studies that have used military participants with PTSD, only one has looked specifically at nightmare content. Freese et al. (29), investigated whether the replicability of nightmares varied according to disorder. The authors compared the nightmares of people with PTSD, depression, or adjustment disorder with those of people with other disorders. The analysis of nightmare content generated the following five subscales (correspondence with reality, reorientation, physiological experience, emotional experience, and dream recall). The results showed that there was a significant association between diagnosis and type of nightmare (χ² (4) = 32.3, p = <.001) and that patients with PTSD had significantly more replicative nightmares than the other disorder groups. The results of this study are interesting insofar as the replicability of nightmares can potentially resemble traumatic events. With this in view, the results of the analyses showed that replicative nightmares generated significantly more distress than other types of nightmares. Moraczewski and McCall (30) have studied nightmare emotional content in a small sample (n=20) of patients with PTSD, showing in particular that anger in nightmares was the best predictor of worsening overall PTSD symptoms. Possemato et al. (31) conducted a similar study among 76 combat veterans with PTSD and at-risk alcohol use. Their results showed that participants whose dreams were highly reproduced and more realistic had significantly higher PTSD intensity scores than those whose dreams were less reproduced and less realistic.

Ansbjerg et al. (32) carried out their study on a trauma-affected refugee population diagnosed with PTSD. This is the only eligible study on a refugee population. Other studies have investigated this population but without a proven diagnosis of PTSD. The authors compared their sample of participants with PTSD (n=20) to a control group (n=20) to investigate the occurrence of sleep pathologies. The results showed that participants with PTSD reported significantly more nightmare frequency than the control group, but that this frequency was very high, with 75% of them having nightmares every day. Belleville et al. (33), conducted an interesting study on participants who had suffered sexual abuse and had PTSD (n=44). In addition to assessing the number of nightmares over a month and the number of nights with nightmares, the authors also measured the associated distress. These evaluative determinants were related to the elements of sexual abuse (age at the time of aggression, familiarity with the aggressor, etc.). The regression analysis results showed that the number of perpetrators was a predictor of the frequency of nightmares (β: 0.34; p.<0.05). Age at the time of sexual abuse explained significantly 9.9% of the variance in distress associated with nightmares. This study is one of the few to have linked specific elements of the probable origin of PTSD with nightmares. Friedmann et al. (34) conducted their research on a PTSD population of women after childhood abuse (n=117), comparing data with mentally healthy women with a history of childhood abuse (n=31) and a control group (n=37). The PTSD group had significantly more nightmares. Mader et al. (27) compared participants who had all experienced a traumatic event, but distinguished between those with a diagnosis of PTSD and those without, and found similar results. Participants with PTSD had significantly more nightmares, and PTSD diagnosis was significantly predicted by posttraumatic nightmare frequency but not by nontraumatic nightmares.

Nightmares are associated with other sleep disturbances, particularly insomnia. Short et al. (35) investigated the predictive factors of insomnia and nightmares in a small sample of 30 participants with PTSD: daily PTSD and fear of sleep predicted a significant increase in nightmares. Nightmares were also predictive of poorer sleep efficiency. Dysfunctional sleep beliefs and insomnia-related behaviors were not associated with nightmares.

PTSD is reported to be a disorder with severe behavioral complications. A quality study carried out on a sample of 255 participants (36) showed that the severity of nightmares had a mediating role in the relationship between PTSD and self-injurious behavior. Suicidal risk is also reported to be present in both PTSD and nightmare sufferers. Very few studies have investigated these interrelationships. Littlewood et al. (37) have contributed to the understanding of these relationships through a study of 91 participants. This quality study examined the basis of the relationship between nightmares and suicidal risk in PTSD through hopelessness, entrapment, and defeat, showing that this variable mediated the relationship between nightmares and suicidal risk. The results also showed that nightmares explained 12.8% of the variance in suicidal behavior.

Among the studies that included participants with PTSD disorder, Ramirez et al. (38) showed that patients hospitalized in psychiatric hospitals during the COVID-19 pandemic had more nightmares than those hospitalized before the pandemic. Van Shagen et al. (39) investigated whether patients with various psychiatric disorders (n=498) show increased symptomatology and different coping styles if they suffer from a comorbid nightmare disorder. Results showed that patients suffering from nightmare disorder had higher psychopathology and personality pathology scores, but no significant differences were found in coping strategies. However, the results of these two studies cannot be applied specifically to PTSD.

### Depressive disorder

10 selected studies conducted their research on a population with a depressive disorder but only one specifically used a cohort of depressed patients.

The majority of studies that have evaluated nightmares in depression disorder have investigated the nature of their relationship with suicide. Depressed patients suffering from nightmares showed significantly higher suicide (40). Tae et al. (41) investigated the suicidal risk of sleep disturbances, including nightmares and bad dreams, in depressed patients. The study was carried out on a large sample of 909 participants, 54.1% of whom were women without other comorbid disorders such as bipolar disorder or psychotic disorder. A regression analysis of this cross-sectional study showed that the occurrence of bad dreams was a significant predictor of suicidal ideation (β: 0.11; p.<0.01). Another study (42) proposed a re-examination of established risk factors for suicidal ideation using quantile regression on a sample (n=354) of psychiatric patients, including depressives. This statistical approach revealed a significant but moderate effect of nightmares (R_2_= 10.8) and showed that the strength of the association between nightmares and suicidal ideation increased with the severity of suicidal ideation. Higher frequency of nightmares was associated independently with male depression and suicide risk as well as in patients who reported monthly to weekly nightmares, impairment due to nightmares was positively associated with male depression (43). Some factors underlie the relationship between nightmares and suicidal risk in depression, such as ruminations. A study (44) carried out on 492 psychiatric patients, 24% of whom had depression, showed that nightmares mediated the relationship between ruminations and suicidal risk. Rufino et al. (45) studied whether difficulties in emotion regulation are associated with nightmares and suicide attempts in a large sample of hospitalized patients (n=2683) (whose percentage of patients with depression is unknown), showing that an association between nightmare frequency and suicide attempts was strongest at high levels of emotion regulation difficulties.

The other studies in the selection were not specifically conducted on a population with a depressive disorder but with a sample including some patients with depression. Attachment styles to the presence of nightmares were investigated and showed that patients with a fearful attachment style reported more difficulties falling asleep due to fear of nightmares (46).

Health contexts are also of interest in understanding nightmares and depression since patients admitted to psychiatric hospitals during COVID-19 had more nightmares than in the pre-COVID-19 period (38).

Psychiatric patients, and in particular those suffering from depression, have more maladaptive personality functioning in terms of self-control, identity integration, responsibility, and social congruence as reported in the study by Van Schagen (2016).

Some studies have investigated the difference in nightmare expression depending on the disorder. Patients with PTSD experience significantly more replicative nightmares than depressed patients (29). In Marinova’s study (40), patients with depression had a higher frequency of nightmares than bipolar patients.

### Anxiety disorder

8 studies have investigated the interrelationship between nightmares, bad dreams, and anxiety disorders, of which only 2 involved a population consisting solely of participants with anxiety disorders.

Nightmare content is linked to anxiety. Some studies (n=2) have used a dream diary to assess and measure bad dreams and nightmares. The use of dream diaries led to thematic analyses. One study (47), carried out on 32 patients with anxiety disorders such as generalized anxiety disorder, social anxiety disorder, panic disorder, agoraphobia, separation anxiety disorder, and specific phobia and in comparison with a control group, showed that the dreams of anxious patients contain significantly more aggressive and sexual interactions, fewer friendly interactions as well as more negative emotions. In the same perspective and with the same cohort, the authors also carried out qualitative analyses (48) on the content of dreams and nightmares of anxious patients who had more threatening content. In addition, the anxiety disorder group reported more nightmare-related distress than the control group.

As with depression, anxiety disorders and nightmares are associated with suicidal risk (43). In a study sample where over 20% of participants (n=492) had an anxiety disorder, nightmares appeared to mediate the relationship between rumination and the presence of a past suicide attempt (44). The underpinnings of the relationship between nightmares and suicidal risk in anxiety disorders can also be explained by emotional regulation difficulties, which appear to moderate the relationship between the frequency of nightmares and previous suicide attempts (45). Moreover, this association between nightmares and suicidal ideation increases with the severity of suicidal ideation (44).

The health context plays a role in the occurrence of nightmares in anxiety disorders since patients, including those with anxiety disorder diagnoses, admitted to psychiatric hospitals during the COVID-19 pandemic had significantly more nightmares than those admitted before the pandemic (38).

Attachment style appears to be a small but significant factor in the frequency of nightmares in a population composed in part of participants with anxiety disorders (46).

### Bipolar disorder

A total of 4 studies were conducted on a population with bipolar disorder. Only one study focused exclusively on this pathology.

Bipolar disorder seems to have links with nightmares, according to its different types (Type I and Type II). In an interesting sample of 141 patients with bipolarity type I and 78 participants with bipolarity type II, data on nightmares were compared with a control group in a cross-sectional study (49). The 20-item NEQ, which measures nightmares across 4 dimensions (physical effect, which reflects a health deficit, negative emotion, interpretation of meaning, and horrible stimulation) was used as the sole measure of nightmares. Patients with all types of bipolarity scored significantly higher than controls on the NEQ, but there was no difference in the frequency of nightmares. The frequency of nightmares was also found to be significantly lower in patients with bipolar disorder than in a group with depression (40). However, high nightmare frequency was positively associated with suicidal risk in a 30% psychiatric sample of patients with bipolar disorder (I and II) (43). Finally, in bipolar disorder too, the influence of the health context (COVID-19 pandemic) is associated with the occurrence of nightmares (38).

### Schizophrenia and psychotic disorder

6 selected studies evaluated nightmares in a population with schizophrenia, schizophrenic spectrum disorder (SPD), or psychotic symptoms. The majority of studies (n=5) have been carried out on a sample composed solely of these disorders. A very interesting study (50), since it was the only longitudinal study among the selection to investigate the prevalence of insomnia and nightmares, as well as their association with suicidal risk, over 8 years in participants (n=388) with SPD. These results highlighted that frequent nightmares were found to be associated with comorbid physical illness, and with a lifetime history of suicide attempts. Nightmare frequency and suicide are therefore also associated with the expression of psychotic pathologies (43). Furthermore, when comparing the frequency of nightmares in schizophrenic patients with high-risk psychotic patients and with a control group, the frequency of nightmares is significantly higher in schizophrenic patients (51).

Insomnia and nightmares are closely associated with schizophrenia. These links are highlighted both by cross-sectional studies using standardized scales (50, 52) and by qualitative studies in which patients are directly questioned during a 90-minute group interview about the phenomenology of their sleep problems (53, 54).

### Suicide

Many studies (n=10) have looked at the links between nightmare markers and suicide (suicidal risk, suicidal ideation, suicide intent, behavior, and acting out). Some of them have studied these links in the context of psychiatric pathologies such as thymic disorders(n=7), PTSD (n=1), psychotic disorders (n=2), or personality disorders (n=2) (see results for PTSD, depression, bipolar disorder, anxiety disorders, psychotic disorders and personality disorders). 2 studies focused on a population at high risk of suicide.

Through a short-term longitudinal study (3 days) on a sample of 91 participants with high suicidal ideation, operationalized by a score of 11 or more on the Beck Scale for Suicidal Ideation, Rogers et al. (55) found that suicide-specific rumination was significantly associated with nightmares and that nightmares were positively related to suicidal intent. Suicide-related cognitions are also associated with nightmares. On transdiagnostic sample of 102 at-risk young completed a 21-day ecological momentary assessment protocol. At the within-person level, nightmares predicted passive suicide ideation, and nightmares, were associated with passive suicide ideation (56).

### Personality disorder

Very few studies (n=3) have examined the links between nightmares and personality disorders. None has used a sample composed exclusively of participants with a personality disorder. Furthermore, in none of the studies did we have any information concerning the type of personality disorder or its cluster.

2 studies have looked at the link with suicidal risk. These samples did not consist solely of participants with a personality disorder. In one study (43), 6.4% of participants had a personality disorder. The results showed that patients hospitalized following a suicide attempt had more weekly nightmares. Another study (45), again with a partial personality disorder population, showed that emotion regulation difficulties were a moderator of the relationship between nightmare frequency and previous suicide attempts.

Attachment style (secure, fearful, and dismissive) is related to nightmare occurrence reports on a study of 2876 psychiatric patients, 33% of whom had a personality disorder (46).

### Attention deficit hyperactivity disorder

Only one study (57) investigated nightmares in a cohort of adult patients with attention deficit hyperactivity disorder (n=65). Patients with ADHD had significantly more nightmares than the control group. In addition, a regression analysis showed that the diagnosis of ADHD was a significant predictor of nightmare frequency (β= 0.17; p.<0.001).

### Nightmare disorder

Studies focusing on a population with a diagnosis of nightmare disorder are scarce (n=2).

Hochard et al. (58) investigated stress tolerance in 53 patients who met the criteria for nightmare disorder according to their Disturbing Dream and Nightmare Severity Index (DDNSI) scores. Stress tolerance was measured using the Reinforcement Sensitivity Theory Behavioral Inhibition System Anxiety subscale. In addition to the DDNSI, a nightmare recall questionnaire was administered on the night before the study. The results showed that participants with nightmare disorder had significantly less resistance to stress than the control group.

Another study (59) looked for differences in personality disorder functioning styles between healthy volunteers (n=219) and patients suffering from nightmares (n=118) showing that patients with nightmare disorder scored significantly higher than healthy volunteers for paranoid, schizotypal, borderline, histrionic, narcissistic, avoidant and dependent styles.

## Discussion

This systematic literature review highlights the close links between nightmares and the psychopathological sphere. Indeed, previous reviews (21, 60) have highlighted this occurrence, particularly in children (61), but have focused on a few specific psychopathological disorders. We wanted to investigate a wider spectrum of pathologies, to reflect the polymorphous expression of the nightmare-psychopathology relationship. In addition, we focused on studies dealing specifically with the expression and repercussions of nightmares in their interrelationships with manifest patterns of psychopathological disorder. To this end, we did not include studies examining these links in the context of therapeutic management of nightmares. In addition, we did not include studies on children and adolescents, as nightmares are more frequent in this population (61). The interoperability of nightmares with psychopathological symptoms and disorders has been put forward in numerous studies (4, 62, 63). The literature emphasizes that the occurrence of nightmares and their repercussions are greater in clinical populations than in the general population (59, 64). However, a certain proportion of these studies have been carried out on a general population (65, 66) likely to present psychopathological symptoms. We have therefore limited the selected studies to a proven clinical population, to give a clearer picture of the association between bad dreams, nightmares, nightmare disorders, and psychopathological symptoms and disorders. Finally, we have only included studies published in the last decade, to reflect the proportion of investigations carried out.

The results of this systematic review underline a first point: nightmares seem to have a privileged relationship with multiple disorders. We found associations between nightmare frequency and distress in post-traumatic stress disorder, depression, bipolar disorder, anxiety disorders, suicidal risk, psychotic disorders, attention deficit hyperactivity disorder, and personality disorders. In our selection strategy, we also focused on pathological bereavement, given its association with sleep disturbance (67). In addition, this type of disorder is likely to be related to post-traumatic stress disorder (68), and therefore to have an important relationship with nightmares. Given our inclusion criteria, we did not retain any study, although some research (69), carried out in the context of bereavement but of a non-pathological nature, has verified an association between the frequency of nightmares and bereavement. We have also looked for studies linking bad dreams, nightmares, and somatoform disorders. Indeed, Faccini et al. (20) showed that nociception was a mediator between nightmare frequency and nightmare-related distress. Despite results suggesting a link between pain and nightmares, we found no studies investigating these links in a population suffering from somatoform disorders.

On the other hand, this relationship seems to be bidirectional, since people with nightmare disorder also have psychiatric comorbidities (70). This bi-directionality, and the wide range of psychopathological disorders associated with nightmares, testify to the disruptive role nightmares play in adult psychic homeostasis. Its association with other nightmare disorders, such as insomnia, is also deleterious in the disruption of psychic equilibrium. Several research studies have investigated the interrelationship between sleep and psychiatry through the two markers of insomnia and nightmares (28, 50, 54). The links between psychopathology and nightmares can be examined in light of the role of dopamine involved in sleep regulation. Indeed, studies have shown the involvement of dysregulation in dopamine receptors in people affected by nightmares (71, 72). on the other hand, adversity in life is associated with striatal dopamine dysregulation and increased cortisol levels in response to a psychosocial situation (73). Furthermore, a recent theoretical model proposes that the propensity for nightmares is due to greater reactivity to internal and external stimuli, and greater depth of cognitive and emotional processing (74, 75). This high reactivity to external stimuli, but also to interoceptive signals, is common to mental pathologies (76, 77), particularly in anxiety and thymic disorders. Moreover, altered interoception has been reported to be associated with mental disorders (78) and nightmares (79). The selected studies, although relevant to the field, did not involve these considerations.

Studies have often highlighted a relationship marked by suicidal risk. Indeed, in many psychopathological disorders, the presence of nightmares is associated with a higher risk of suicide. The strength of the association between nightmares and suicide was also compared across pathologies. Marinova et al. (40) found that patients suffering from depression had more nightmares than those with bipolar disorder. However, certain studies (43, 44) provide convincing information on these interrelations, but these studies are carried out on several psychiatric populations without shedding any light on the singularity of the relationship between nightmares and a specific psychopathological disorder. This is a major shortcoming, in general, and beyond the understanding of suicidal risk, particularly in understanding the expression of nightmares in personality disorders, where no personality disorder has been distinguished (45, 46) although these studies bring relevant elements to the scientific literature. Yet common patterns between suicidality and nightmares may be relevant to increase the interest of these studies. The dopaminergic pathway, as mentioned earlier in the discussion, appears relevant to understanding the underpinnings of this relationship. Indeed, the literature shows that dopaminergic disturbances play a role in the biology of people who have attempted suicide (73, 80).

Our selection criteria included studies on a population suffering from nightmare disorders. Very few studies have examined this disorder as it relates to psychopathological symptoms. Yet, given the bidirectional links between nightmares and psychopathology, the study of the patterns underlying this disorder and its evolution would constitute important elements for the literature.

The results of this systematic review highlight another gap in research: the small number of longitudinal studies. Only 3 studies (50, 55, 56) were selected, including 2 short-term (21 days and 3 days) longitudinal studies. Some studies (have used a partial long-term method (26, 34) (collection of nightmare data but not of psychopathological symptoms), notably through the use of daily dream/sleep diaries. The literature suggests that nightmares may be a prodromal symptom in the onset of psychopathological disorders (81). Moreover, it is associated with the severity of the illness. Longitudinal studies are of great value in clarifying the nature of nightmares. In addition, studies exploring the links between nightmares, bad dreams and psychopathologies may focus on understanding the underpinnings of this relationship, notably through a neurochemical prism, but also by testing theoretical models of nightmares. Understanding these underlying processes is crucial to the deployment of medicinal and non-medicinal therapies, both for psychiatric disorders and for nightmares as a transdiagnostic process.

Overall, our results clearly show an important relationship between bad dreams, nightmares, and psychopathology. These data have implications for clinical practice. Studies have shown that the treatment of nightmares has a positive influence on the improvement of psychological symptoms (82). It is still unclear to what extent early treatment of nightmares can stem the onset or development of psychopathological disorders.

All in all, the field of research into nightmares and psychopathological disorders remains poor. The literature can attest to a bidirectional relationship between nightmares and psychopathology, but the complexity of these links is still largely unknown. Moreover, as pointed out earlier, certain pathologies such as depression and post-traumatic stress disorder are benefiting from more studies to clarify their relationships. Many other pathologies, such as psychotic disorders or personality disorders, notably borderline personality disorder, deserve particular attention. A better understanding of how nightmares are expressed in these high-risk suicide disorders could help to maximize therapeutic efforts and tools through early detection, prevention, and treatment of nightmares.

This systematic literature review has limitations. Firstly, we only included studies with a clinical population, thus limiting our perspective on the deleterious force of nightmares. We have not strictly observed PRISMA standards, which may lead to flaws in the literature review. The included studies were not pre-registered, which may induce publication bias. We did not provide information on the drug treatments of the populations studied, although some drugs do have an impact on the expression of nightmares. Although we only selected studies carried out on a population under the age of 60, we were unable to determine whether the selected studies had not included participants with diseases such as epilepsy, sleep apnea, migraine or neurodegenerative diseases such as early-onset Parkinson’s disease, which could also explain the occurrence of nightmares.

Given our results, future studies should be based on a longitudinal methodology to confirm the prodromal nature of bad dreams and nightmares. Other pathologies, such as personality disorders, ADHD or schizophrenia spectrum disorders, will need to be investigated to complement the current data in the scientific literature. Finally, given the importance of the links between suicide and nightmares in multiple pathologies, this link should be investigated more specifically, notably through the prism of the dopaminergic pathway.

In conclusion, this systematic literature review has provided a more exhaustive account of the psychopathological disorders affected by nightmares. We have also highlighted the fact that studies on the interrelationships between bad dreams, nightmares, and psychopathology are still too few and that this field of study offers important perspectives for the clinic.

## Data Availability

The raw data supporting the conclusions of this article will be made available by the authors, without undue reservation.
