# Supplementary material for: Bad dream, nightmares and psychopathology: a systematic review
Source: Front Psychiatry. 2024 Oct 8;15:1461495. doi: 10.3389/fpsyt.2024.1461495 (PMC11493664; doi:10.3389/fpsyt.2024.1461495)
Supplement: Supplementary file 1 [file Table1.docx]

| **Author, years**  Supplementary Table 1 : Summary of studies. | **Country** | **Sample** | **N** | **Average Age, Sex F(%)** | **Study objectives** | **Measures of Nightmares** | **Measures of Psychiatric symptoms/others** | **Main Results** | **Comments** | **Quality score** |
| --- | --- | --- | --- | --- | --- | --- | --- | --- | --- | --- |
| Ansbjerg et al., 2023 | Denmark | Refugee with Post-Traumatic Stress Disorder | 40 (20 patients/20 controls) | Patients: 42.21, 25%  Control: 42.67, 25% | To examine subjective sleep quality, measure sleep architecture, and identify latent sleep disorders in refugees diagnosed with PTSD compared to controls | The Disturbing Dream and Nightmare Severity Index (DDNSI) | .The Harvard Trauma Questionnaire (HTQ)  The Hopkins Symptom Checklist 25 (HSCL-25)  .The Hamilton Anxiety and Depression Rating Scale  . Polysomnography | Patients have significantly higher DDNSI scores than the control group (HCs).  75% of the patients reported weekly nightmares compared to 1% of HCs. 70% of the patients reported that their nightmares were of a severe intensity compared to 0% of HCs. |  | 16 |
| Freese et al., 2018 | Germany | Members of the armed forces  treated in psychiatric centers for post-traumatic stress disorder (PTSD), depressive disorders or adjustment disorders. | 127 | 30.5 ; 16.5% | To test whether the degree of replicability to which nightmares resemble potentially traumatic events varies among different disorders. | .Hamburg Nightmare Questionnaire for Military Personnel (HNMP)  . Analysis of dominant nightmare type (Nightmare realism, the degree to which the nightmares  nightmares had replicative, non-replicative or mixed content and correspondence with the HNMP. | .General Depression Scale  . Impact of Event Scale-Revised (IES-R)  . | . Significant association between diagnosis and nightmare type.  . Patients with PTSD experienced significantly more replicative nightmares than patients with a depressive episode or adjustment disorder.  . Patients with an adjustment disorder experienced significantly more non-replicative nightmares than patients with a depressive disorder or PTSD.  . Subjective distress caused by nightmares was significantly more pronounced in the case of replicative nightmares. |  | 14 |
| Mäder et al., 2023 | Switzerland | Pparticipants who had suffered a DSM-5-indexed trauma(various types of trauma) in the last 2 years with or without PTSD diagnosis. | 122  PTSD (59)  No PTSD (63) | 24.16 ; 68% | Study the relationship between psychophysiological measures during awakening and the diagnosis of PTSD,  post-traumatic nightmares and non-traumatic nightmares. | . Nightmares log (14 consecutive days) | . Clinician-Administered PTSD Scale for DSM-5 (CAPS-5)  . PTSD Checklist for DSM-5 (PCL-5)  . Acoustic startle paradigm  . Measures of e heart rate (HRR), skin conductance (SCR), orbicularis oculi electromyogram (HRV) | . Significant positive associations between PTSD symptom severity and posttraumatic nightmare frequency.  . Post-traumatic nightmare frequency was significantly higher for participants with a PTSD diagnosis.  . Nontraumatic nightmare frequency was not significantly correlated with the PCL-5 score.  . PTSD diagnosis was significantly predicted by posttraumatic nightmare frequency but not by nontraumatic nightmares.  . Significant positive association between posttraumatic nightmare frequency and the HRR.  . Nontraumatic nightmare frequency was significantly correlated with the SCR.  . HRR significantly predicted posttraumatic nightmares but not nontraumatic nightmares. |  | 16 |
| Friedmann et al., 2022 | Germany | Women with PTSD after childhood abuse (CA) + mentally healthy women with a history of CA + Control group (CG) | PTSD : 117  CA : 31  GC : 37 | PTSD  37.3 ; 100%  CA  34.5 ; 100%  CG  30.3 ; 100% | Evaluation of sleep parameters of women with PTSD and emotional instability after CA compared with two groups of mentally healthy women with and without CA experience. | Daily (6 days) : sleep time, number of nightmares, sleep quality.  . Pittsburgh Sleep Quality Index (PSQI) | . Structured Clinical Interview for DSM-IV (SCID)  . Global Assessment of Functioning Scale (GAF)  . BPD section of the International Personality Disorder Examination (IPDE)  . Brief Symptom Inventory (BSI)  . Beck Depression Inventory-II, BDI-I  . Quality of life (WHOQOL-BREF)  . Trauma history was assessed via the Life Events Checklist for DSM-5 (LEC-5)  . PTSD Checklist for DSM-5 (PCL-5)  . PTSD Scale for DSM-5 (CAPS-5) | . The PTSD group reported lower sleep quality, more nights with nightmares, and shorter sleep duration than both the CA group and CG.  . The PTSD group reported having trauma-related nightmares in 36% of the nights, whereas participants in the HTC and HC groups experienced nightmares in 2 and 6% of the nights. |  | 20 |
| Possemato et al., 2022 | United States | Participants with positive screening for post-traumatic stress disorder and alcohol consumption among combat veterans. | 76 | 31 ; 10% | Analysis of nightmare characteristics about PTSD symptom severity | . Coding of nightmares according to intensity and type of emotion (fear, shame, anger, sadness) on the Clinician-Administered PTSD Scale (CAPS) | . Clinician-Administered PTSD Scale (CAPS)  . Alcohol Use Identification Test (AUDIT) | . Participants with highly replicating and more realistic dreams had significantly higher PTSD intensity scores than those with less replicating and less realistic  dreams. |  | 13 |
| Miles et al., 2022 | United States | Active-duty service members or recently discharged veterans diagnosed with PTSD assessed by the Clinician Administered PTSD Scale for DSM-5 and diagnosed  with insomnia disorder and nightmare disorder assessed by the  Structured Clinical Interview for DSM-5 Sleep Disorders. | 93 | 36.25 ; 27% | Characterizing associations between sleep disorders and symptoms of post-traumatic stress disorder (PTSD) | . Consensus Sleep Diary for 7 days | . Insomnia Severity Index (ISI)  . Polysomnography  . PTSD Checklist for DSM-5 (PCL-5)  . Dimensions of Anger Reactions-5 (DAR-5)  . Polysomnography | . A higher average frequency of nightmares and greater severity of nightmares  were significantly associated with greater post-traumatic stress symptoms.  . Greater nightmare severity was significantly  associated with greater anger symptoms. |  | 20 |
| Miller et al., 2018 | United States | Male United States military veterans engaged in residential treatment for PTSD. | 31 | 41.23 ; 0% | Understanding disruptive nightmares and dreams in post-traumatic stress a using intensive, longitudinal, and ambulatory methods. | . Diary app (6 weeks) : measuring the presence of nightmares (NM) or disturbing dreams (DD) + intensity of emotions. | . CAPS-5  . Structured Clinical Interview  . Severity of traumatic brain injury (TBI)  . Mattress actigraphy  . Obstructive sleep apnea risk estimation (REI) | . A greater likelihood of a morning NM/DD report was associated with elevated REI  . Elevated REI and lower prior-night sleep respiratory sinus arrhythmia predicted morning endorsement of NM/DD |  | 16 |
| Short et al., 2018 | United States | Individuals with PTSD diagnosis assessed by clinical interview | 30 | 38.03; 61.3% | Test both PTSD-specific and insomnia-specific predictors of sleep quality, efficiency, and nightmares in a sample of PTSD patients. | Disturbing Dream and Nightmare Severity Index (DDNSI) | . Structured Clinical Interview for DSM-5 (SCID)  . PTSD Checklist (PCL-5)  . Insomnia Severity Index (ISI)  . Beck Anxiety Inventory (BAI)  . Beck Depression Inventory-II (BDI-II)  . Fear of Sleep Inventory (FOSI)  . Dysfunctional Beliefs and Attitudes about Sleep Scale (DBAS)  . Sleep-Related Behaviors Questionnaire (SRBQ) | . PTSD, daily PTSD, and fear of sleep predicted increased nightmares significantly.  . Anxiety, depression, and daily nightmares continued to predict poorer sleep efficiency significantly. |  | 16 |
| Moraczewski et al., 2019 | United States | Patients seeking routine outpatient care with diagnosis of PTSD and who had a Disturbing Dreams and Nightmares Severity Index (DDNSI) score of greater than 10. | 20 | 39.8 ; 85% | To examine the affective characteristics of nightmares in 20 patients with post-traumatic stress disorder. | .Visual-analogue scale(VAS) to quantify the frequency of various aspects of the patient’s dreams and were rated from “0” (never) to “100’ (always).  . Nightmares Severity Index (DDNSI) | . SCID  . CAPS Baseline. Hamilton Rating Scale for Depression (HRSD)  . C-SSRS  . Clinical global impression-severity (CGI-S)  . Insomnia Severity Index (ISI)  . PTSD checklist-specific version (PCL-S) | . Dreams were reported to be frequently frightening or sad.  . The presence of anger was positively correlated with PCL score  . PTSD nightmares are negative affect-laden, and specifically, anger in dreams is correlated with a greater degree of traumatic themes, repetitiveness, and awakenings.  . Anger in dreams best indicated the presence of worse global PTSD symptoms. |  | 18 |
| Belleville et al., 2019 | Canada | Victims of sexual abuse with post-traumatic stress disorder. | 44 | 30.73  88.6% | Determine whether abuse-related characteristics predicted sleep disturbances in a sample of sexual abuse victims suffering from PTSD and sleep disturbances. | Nightmare Distress Questionnaire (NDQ)  . No. of nightmares per month  No. of nights with nightmares per month | .Clinician Administered PTSD Scale (CAPS)  .Pittsburgh Sleep Quality Index (PSQI)  . Modified PTSD Symptom Scale – Self Report (MPSS – SR) | . The NDQ total score was correlated with age at the time of sexual abuse and with victim-perpetrator relationship; participants who reported abuse before the age of 18 and perpetrated by a family member or a partner (had more distress associated with the nightmare.  . Nightmare frequency was correlated with the number of perpetrators.  . Age at the time of sexual abuse was statistically significant |  | 18 |
| Littlewood et al., 2016 | England | Participants who had experienced trauma including patients with current diagnosis of PTSD | 91  PTSD : 50 | 28.87 ; 73% | . Investigate whether nightmares were associated with suicidal behaviors.  . Examine possible multistep indirect pathways of the association between nightmares and suicidal behaviors through (i) defeat, (ii) entrapment, and (iii) hopelessness. | . Nightmare severity: 2 recurrent or distressing dreams items within the Clinician-Administered PTSD Scale for DSM-IV (CAPS) | . Suicidal Behaviors Questionnaire-Revised (SBQ-R)  . Defeat: 16-item scale  .Entrapment: 16-item scale  . Beck Hopelessness Scale (BHS)  . Insomnia: 2 difficulty in falling or maintaining sleep items within the CAPS | . Nightmares significantly predicted suicidal behaviors, independent of insomnia.  . Nightmares were both directly and indirectly associated with suicidal behaviors, through perceptions of defeat, entrapment, and hopelessness, independent of comorbid insomnia and depression. |  | 21 |
| Short et al., 2015 | United States | Patients presenting for outpatient mental health services with ¨PTSD or depressive disorder diagnosis according to Structured Clinical Interview for DSM-IV. | 255 | 26.38; 65.1% | To test the hypothesis that post-traumatic stress disorder, but not other anxiety-related disorders, would be associated with self-injurious behavior,  after covariation with depression. | . Disturbing dreams and nightmares severity index (DDNSI) | . Insomnia severity index (ISI)  . Self-injurious behaviors: SCID-II  . Ruminative response scale (RRS)  . Structured clinical interview for DSM-IV Axis I disorders—patient edition (SCID-I-P) | Nightmare severity mediated the relationship between PTSD and self-injurious behavior. |  | 20 |
| Tae et al., 2019 | Republic of Korea | Patients seeking treatment with a diagnosis of depression disorder with or without suicidal ideation. | 909 | 35.81 ; 54.1% | To examine the relationship between sleep problems and suicidal ideation in depressive  patients beyond the effect of depressive symptoms | . The Pittsburgh Sleep Quality Index (PSQI) | . Beck Depression Inventory (BDI)  . Suicidal ideation (Item-9 BDI) | Bad dreams have been associated with an increased risk of suicide. |  | 21 |
| Ma et al., 2018 | China | Healthy volunteers and patients with Bipolar Disorder I (BDI) and Bipolar Disorder II (BDII) | .Healthy volunteers (200)  .BD I (141)  .BD II (78) | Healthy volunteers :  20.06 ; 60.5%  BDI: 20.15 ; 63.12%  BDII : 20.26 ; 65.38% | Determine whether the frequency of nightmares or their content are different in BD I and BD II. | . . Nightmare Experience Questionnaire NEQ) | . Mood Disorder Questionnaire (MDQ)  . HCL-32 (hypomanic symptoms)  .  **Plutchik**-van Praag Depression Inventory (**PVP**) | . Patients (BDI and BDII) scored significantly higher than controls on the NEQ  . No significant difference was identified among the three groups regarding the nightmare frequency  . NEQ Physical Effect was significantly and positively correlated with MDQ. |  | 12 |
| Reed & Rufino, 2019 | United States | Psychiatric patients with a diagnosis of mood disorder, anxiety disorder, substance use disorder, or personality disorder. | 2876 | 34.88; 47.7% | Explore the relationships between adult attachment style, nightmare frequency, disturbed sleep due to nightmares, and difficulty falling asleep due to fear of nightmares in psychiatric Inpatients. | . The Sleep-Disturbance Screening (SDS) | . The Relationship Questionnaire  . The Big Five Inventory  . The Stressful Life Events Screening Questionnaire | . Small significant associations between nightmare frequency and secure, fearful, and dismissing attachment styles.  . Small significant associations between disturbed sleep due to nightmares and fearful, dismissing, attachment styles.  .Patients with a fearful attachment style reported significantly more difficulty falling asleep due to fear of nightmares than patients with secure, preoccupied, or rejecting attachment styles.  . Fearful attachment style and a history of trauma were more predictive of sleep disturbances due to nightmares than trait neuroticism alone. |  | 17 |
| Rogers & Joiner, 2017 | United States | Psychiatric outpatients (major depressive disorder ((22%), social anxiety disorder (12.1%), generalized anxiety disorder (7.6%) substance use disorders (7.3%) | 354 | 27.01 ; 61.3% | Re-examine established risk factors for suicidal ideation using quantile regression. | . Disturbing Dreams and Nightmares Severity Index (DDNSI) | . Interpersonal Needs Questionnaire (INQ)  . Brief Agitation Measure (BAM)  . Insomnia Severity Index (ISI)  . Anxiety Sensitivity Index – Cognitive Concerns Subscale (ASI-CC)  . Ruminative Responses Scale – Brooding Subscale (RRS)  . Beck Scale for Suicide Ideation (BSS) | . Nightmares have a moderate effect on suicidal ideation (R₂= 10.8)  . The strength of the association between nightmares and suicidal ideation increases with the severity of suicidal ideation. |  | 18 |
| Rufino et al., 2020 | United States | Psychiatric inpatient with multiple comorbid conditions, prominently mood, anxiety, substance-related, and personality disorders. | 2683 | 34.5 ; 52.5% | Investigate whether emotion regulation difficulties are associated with nightmares and suicide attempts in an inpatient sample. | . Frightening dreams or nightmares each of the past two weeks | . Difficulties in Emotion Regulation Scale (DERS)  . Lifetime number of suicide attempts | . Emotion regulation difficulties as a moderator of the relationship between nightmare frequency and previous suicide attempts  . The association between nightmare frequency and suicide attempts was strongest at high levels of emotion regulation difficulties  . The interaction of nightmare frequency and the DERS scale was significant for each subscale except for difficulties engaging in goal-directed behavior. |  | 15 |
| Ramirez et al., 2022 | United States | Adults admitted to an inpatient psychiatric hospital pre-pandemic or admitted during the COVID-19 pandemic (Major depression disorder, General anxiety disorder, PTSD, bipolar disorder) | 470 | 34.86 ; 44% | Examine symptom severity among psychiatric inpatients admitted prior to versus during the COVID-19 pandemic. | Disturbing Dream and Nightmare Severity Index (DDNSI) | . Generalized Anxiety Disorder Scale (GAD-7)  . Patient Health Questionnaire (PHQ-9)  . Patient Health Questionnaire for Adolescents (PHQ-A)  . Difficulties in Emotion Regulation Scale- Short Form (DERS-SF)  . World Health Organization Disability Assessment Scale (WHODAS)  . World Health Organization Alcohol, Smoking, and Substance Involvement Screening Test (WHOASSIST)  . Pittsburgh Sleep Quality Index (PSQI)  .Suicide Behaviors Questionnaire-Revised (SBQ-R) | . Group admitted during the pandemic had significantly more nightmares  . |  | 22 |
| Lamis et al., 2018 | Italia | Inpatients consecutively hospitalized at psychiatric units. Non-affective psychotic (23.3%), bipolar-I (26.2%) bipolar-II (3.5%), major depressive ( 11.6%), Schizoaffective (23.3%), anxiety disorders; (12.8%), personality disorders (6.4%) | 172 | 39.15; 52.9% | Test the hopelessness theory of suicide risk in psychiatric patients  who may or may not be experiencing nightmares. | Disturbing Dream and Nightmare Severity Index (DDNS) | . Beck Hopelessness Scale (BHS)  . Gotland Male Depression Scale (GMDS)  . Mini International Neuropsychiatric Interview (MINI) | . Patients who reported monthly to weekly nightmares were younger and more likely to have been hospitalized for a recent suicide attempt. They also reported higher scores on the BHS, GMDS, and MINI suicide risk than other patients.  . A higher frequency of nightmares was associated independently with male depression and suicide risk.  . Hopelessness was not significantly associated with the frequency of nightmares when controlling for male depression and suicide risk.  . In patients who reported monthly to weekly nightmares, impairment due to nightmares was significantly and positively associated with male depression. |  | 19 |
| Rogers et al., 2017 | United States | Psychiatric outpatients; major depressive disorder (24%.0), social anxiety disorder (13.0%), generalized anxiety disorder (10.2%), and substance use disorders (10.0%) | 492 | 26.75  64.2% | Test the hypothesis that various types of overarousal (agitation, insomnia, nightmares) would account for the relationship between brooding and both suicidal ideation and attempts. | Disturbing dreams and nightmares severity index (DDNSI) | . Ruminative responses scale e brooding subscale (RRS)  . Brief agitation measure (BAM)  . Insomnia severity index (ISI)  . Beck scale for suicide ideation (BSS) | . The indirect relationship between brooding and suicidal ideation through nightmares was significant in a model that explained 15.82% of the variance in suicidal ideation.  .Nightmares significantly accounted for the  association between brooding and suicidal ideation.  . Nightmares mediator the relation between brooding and the presence of a past  suicide attempt. |  | 20 |
| Marinova et al., 2014 | Bulgaria | Inpatients meeting criteria for a current depressive episode within Recurrent Depressive Disorder/RD Bipolar Disorder/BD according to the ICD-10. | 52 (44RD ; 8 BD) | Aged from 24 to 75 years | Tested the hypothesis that nightmares are associated with an elevated suicidal risk in depressed patients. | A direct interview about the frequency, content and emotional charge of patients’ dreams. | Suicide risk was measured in item 3 (score 0 to 4) of [Hamilton Depression Rating Scale (HDRS)](https://dcf.psychiatry.ufl.edu/files/2011/05/HAMILTON-DEPRESSION.pdf) | . Significant difference in the proportion of patients with troubling nightmares: 64% of the RD patients had nightmares and only 25% of the BD patients.  . The two subgroups did not differ significantly in their suicide risk.  . In the RD subgroup, the patients with nightmares had significantly higher average scores on the HDRS item on suicide risk than those without nightmares. | No information on average age. | 14 |
| Van Schagen et al., 2016 | Netherland | Patients who received psychiatric treatment with a primary diagnosis of mood disorder, anxiety disorder, PTSD, or « other » | 498 | 35.7, 72.1% | Investigate whether patients with diverse psychiatric disorders have increased symptomatology and different coping styles if they suffer from comorbid nightmare disorder | .The Nightmare subscale of the SLEEP-50  . Additional questions :  « “Do nightmares affect your well-being?” “How many disturbing dreams did you have in the last seven days?”; “How many nights with disturbing dreams did you have in the last seven days?”; “How many disturbing dreams did you have in the last month?”  . The Nightmare Distress Questionnaire (NDQ)  . The Nightmare Effects Survey (NES) | . The Symptom Check List (SCL-90)  . Severity Indices of Personality Problems (SIPP)  . Coping Inventory for Stressful Situations (CISS) | . Patients with nightmare disorder had a significantly higher SLC-90 score.  . About personality psychopathology, the nightmare disorder group had significantly  higher scores, indicating more maladaptive personality functioning on self-control, identity integration, responsibility, and social concordance.  . No difference in terms of relational capacities. |  | 18 |
| Rimsh, 2021 | Germany | Outpatients with various anxiety disorders ( generalized anxiety disorder, social anxiety disorder, panic disorder, agoraphobia, separation anxiety disorder, specific phobia + healthy people with no previous or current mental disorder. | Patients: 38  Healthy: 38 | Patients :  28.32 ; 71.05%  Healthy :  30.08 ; 71.05% | To investigate several dream  characteristics of outpatients with anxiety disorders in comparison to dream characteristics of  healthy individuals | . Dream diary (21 days)  .Multidimensional Düsseldorf Dream Inventory (MDTI)  . Mannheim Dream Questionnaire (MADRE)  . The Nightmare Behavior Questionnaire (NBQ)  . Nightmare Distress Questionnaire (NDQ) |  | . Anxiety patients report more  aversive dreams, better dream recall, and more incorporations from waking life into dreams.  Their dreams are rated as having a higher emotional intensity, a lower dream mood, a  higher (retrospective) nightmare frequency, and more nightmare distress report that their mood  is much more often affected by their dreams.  . Nightmares during the study were significantly higher in the group of anxiety patients than in the healthy control group. |  | 15 |
| Rimsh & Pietrowsky, 2020 | Germany | Outpatients with various anxiety disorders ( generalized anxiety disorder, social anxiety disorder, panic disorder, agoraphobia, separation anxiety disorder, specific phobia + healthy people with no previous or current mental disorder. | Patients : 38  Healthy: 38 | Patients :  28.32 ; 71.05%  Healthy :  30.08 ; 71.05% | Investigate specific characteristics manifested in dreams of patients with clinical anxiety disorders and to compare them with dreams of healthy persons. | . Dream diary (21 days) + content analysis ( Characters, Social Interactions, Activities, Success and Failure, Good Fortune and Misfortune, Emotions, Settings and Locations, Objects, Descriptive Elements). |  | The dreams of anxious patients contain significantly more aggressive and sexual interactions, fewer friendly interactions, a higher frequency of failure and misfortune, bad luck and anxiety, unlucky, dangerous, and threatening situations and events, and a lower prevalence of success, fortune, and happy situations, as well as more negative emotions. |  | 15 |
| Cox et al., 2023 | United States | Participants with suicidal ideation (SI) and/or suicidal behaviors Columbia Suicide Severity Rating Scale, Posner  in the 4 months preceding the study. | 102 | 24.92 ; 74.5% | Examine the components of subjective sleep disturbance as predictors of passive and active suicidal ideation at both the  intra-personal (i.e. daily changes within individuals compared with their own mean) and inter-personal (individual differences compared with the sample mean). | . Pittsburgh Sleep Diary (21 days) | . Daily passive and active suicide ideation (21 days)  . Daily depressed mood (21 days) | . A significant positive effect of nightmares on intrapersonal passive SI  . a significant positive effect of nightmares on passive SI at the interpersonal  . Nightmares have no significant effect on active SI at the intra- or inter-personal level | Sample with multiple mental disorders | 19 |
| Rogers et al., 2021 | United States | Participants with elevated suicidal ideation operationalized as a score of 11 or higher on the Beck Scale for Suicide Ideation. | 91 | 27.03 ; 53.8% | To examine the short-term longitudinal (3 days) association between suicide-specific rumination and suicidal intent, controlling for multiple factors in a high-risk suicide sample. | .Items nightmares. Items drawn from the Acute Suicidal Affective Disturbance Inventory—Current/Lifetime versions since the last assessment period. | . Suicide Rumination Scale (SRS)  . Interpersonal Needs Questionnaire (INQ)  . Items assessing agitation, insomnia, and nightmares. Items drawn from the Acute Suicidal Affective Disturbance Inventory—Current/Lifetime versions.  . Depressive Symptoms Inventory – Suicidality Subscale (DSI-SS)  . Suicidal Intent (one item)  . Previous Suicide Plans and Attempts | . Suicide-specific rumination remained significantly associated with nightmares.  . Nightmares and suicidal ideation were positively related to suicidal intent. | No information on participants' diagnoses and treatments | 21 |
| Xin Li, 2016 | Hong-Kong | Psychiatric outpatients with  schizophrenia-spectrum disorders | 388 | 41 ; 54.9% | To examine the prevalence of sleep disorders and their prospective associations with the risk of suicide through an 8-year longitudinal study. | Sleep questionnaire to assess  demographics, lifestyle, sleep habits and specific sleep problems, including insomnia and nightmares and their frequency  frequency over the past year. | . Diagnosed by the attending psychiatrist based on the criteria of the International Classification of Diseases, Tenth Revision (ICD-10 | . Participants who had frequent nightmares were more likely to report frequent insomnia.  . Frequent nightmares were found to be associated with the comorbid physical illness.  . Frequent nightmares were associated with a lifetime history of suicide attempts.  .Participants who reported comorbid insomnia and nightmares at baseline had a greater risk of suicide attempts in the following 8 years. |  | 19 |
| Chiu et al., 2016 | Australia | Participants with a diagnosis of a schizophrenia spectrum disorder (schizophrenia, schizoaffective disorder) | 14 | 18 years of age or older, 50% | To explore the lived experience of sleep problems in people with schizophrenia  schizophrenia through small focus groups and individual interviews. | Focus groups of 2 to 4 participants lasting 60 to 90 minutes to discuss topics related to sleep experience problems.  . Thematic analysis: frequency, movement, specificity, scope, and overview. |  | . Nightmares and fears were closely linked to the maintenance of insomnia.  . Adverse life events have been linked to nightmares and bad dreams.  . The issue of medication was often raised as a factor contributing to nightmares. | No information on the average age of participants.  . Length of illness mean:16.6 years  . Qualitative data only. | 9 |
| Chang et al., 2021 | Taiwan | Participants with a diagnosis of schizophrenia and recruited from three community-based mental health centers. | 18 | 41.39; 50% | Identify factors related to sleep problems and coping methods used by people with schizophrenia. | . Pittsburgh Sleep Quality Index (PSQI)  . Individual interview: questions about sleep experiences, attitudes, and methods for coping with sleep problems | . Pittsburgh Sleep Quality Index (PSQI)  . Thematic analysis  . Individual interview: questions about sleep experiences, attitudes, and methods for coping with sleep problems | Psychiatric symptoms and nightmares were identified as unique sleep disruptions in people with  Schizophrenia. | Qualitative analysis only | 9 |
| Michels et al., 2014 | Germany | Inpatients diagnosed with schizophrenia (SZ) + patients in at-risk mental states for psychosis (ARMS) + healthy relatives of patients (H) | SZ: 17  ARMS : 14  H: 17 | SZ :  32.88 ; 47.06  ARMS :  23.29 ; 35.71%  H :  34.31 ; 58.82% | To investigate nightmare frequency and its correlates in patients with schizophrenia | . Subjective assessment of dream frequency over the past two months on a 7-point scale + frequency of nightmares on a  8-point scale | . Beck Depression Inventory (BDI)  . Positive and Negative Syndrome Scale (PANSS)  . Early Recognition Inventory (ERIraos)  . Erholungs-Belastungs Fragebogen (EBF) | . Nightmare frequency revealed significantly higher frequencies for the patients with SZ and ARMS compared to healthy controls.  . PANSS and nightmare frequency have no significant correlation.  . The frequency of nightmares is related to patients' subjective distress. |  | 16 |
| Sheaves et al., 2015 | England | In-and outpatients who experienced psychotic symptoms | 40 | 41.9, 37.5% | Investigate the phenomenology of nightmares in the context of psychosis. | . A retrospective dream log (14 nights) | . The Pittsburgh Sleep Quality Index  . The Psychotic Symptoms Rating Scale  . The Depression, Anxiety, and Stress Scales  . The Posttraumatic Diagnostic Scale  . The Time Budget Questionnaire  . The Digit Span Subtest | . 42.5% of participants screened positive for PTSD. 65% reported weekly nightmares  . A large positive correlation was found between nightmare frequency and overall (PSQI) sleep quality while controlling for antipsychotic dose.  . Nightmare distress was significantly correlated with 4 measures of daytime psychological functioning: delusional severity, depression, anxiety, and stress. |  | 19 |
| Schredl et al., 2016 | Germany | . Patients with Attention-deficit hyperactivity disorder (ADHD) diagnosis, Outpatient Clinic at the Central Institute of Mental Health. Patients fulfilled the criteria for ADHD according to DSM–IV criteria.  . Control group | 65 | Patients:39.88  47.69%  Control :  48.14  53.74% | Test whether adult ADHD patients show an increased nightmare frequency. | .Eight-point rating scale ; (“How often do you experience nightmares?” 0: never, 1: less than once a year, 2: about once a year, 3: about 2–4 times a year, 4: about once a month, 5: about 2–3 times a month, 6: about once a week, 7: several times a week. |  | . Patients have more nightmares than controls.  . Ordinal regression shows that having an ADHD diagnosis, age, and gender are significant predictors of nightmare frequency. | No quantitative inter-group comparisons. | 18 |
| Hochard et al., 2016 | United Kingdom | Participants with a nightmare disorder (ND)according to the Disturbing Dream and Nightmare Severity Index (DDNSI+ Controls | ND: 43  Controls: 42 | 20.95 ; 85.88% | To investigate the impact of frequent nightmares on a stressful  cognitive test (the PVSAT-C). | . Disturbing Dream and Nightmare Severity Index (DDNSI)  . nightmare recall questionnaire (binary variable: yes/no) assessed for the occurrence of a nightmare on the night before the study. | . Beck Depression Inventory-II (BDI-II)  . Pittsburgh Sleep Quality Index (PSQI)  . Reinforcement Sensitivity Theory Behavioral Inhibition System Anxiety subscale (rRST BIS Anxiety) | . Significant difference between groups in stress tolerance (participants experiencing frequent nightmares had shorter latencies for forcibly exiting the stress task compared to control participants). |  | 17 |
| Shao et al., 2020 | China | . Patients with nightmare disorders according to the DSM-V criteria  . Healthy volunteers | 118 (patients)  219 (healthy volunteers) | Patients:22.82  68.6%  Healthy volunteers :  23.21  68% | Look for the differences in nightmare experiences and personality disorder functioning styles and their interrelationships between healthy volunteers and nightmare disorder patients. | .Nightmare Experience Questionnaire (NEQ)  . Diagnosis by a psychiatrist according to the DSM-V criteria. | . Parker Personality Measure (PERM)  . Plutchik–van Praag Depression Inventory (PVP) | Nightmare disorder patients scored significantly higher than healthy volunteers on all NEQ scales.  .PERM style scores were significantly different between the two groups: patients scored significantly higher than healthy volunteers on Paranoid, Schizotypal, Borderline, Histrionic, Narcissistic, Avoidant, and Dependent styles.  . Nightmare disorder patients scored higher on the PVP score. |  | 17 |
